# Quantum Enhancement of a S/D Tunneling Model in a 2D MS-EMC Nanodevice Simulator: NEGF Comparison and Impact of Effective Mass Variation

**DOI:** 10.3390/mi11020204

**Published:** 2020-02-16

**Authors:** Cristina Medina-Bailon, Hamilton Carrillo-Nunez, Jaehyun Lee, Carlos Sampedro, Jose Luis Padilla, Luca Donetti, Vihar Georgiev, Francisco Gamiz, Asen Asenov

**Affiliations:** 1 Device Modelling Group, School of Engineering, University of Glasgow, Glasgow G12 8LT, UK; 2 Nanoelectronics Research Group, Departamento de Electrónica y Tecnología de Computadores, Universidad de Granada, 18071 Granada, Spain

**Keywords:** direct source-to-drain tunneling, transport effective mass, confinement effective mass, multi-subband ensemble Monte Carlo, non-equilibrium Green’s function, DGSOI, FinFET

## Abstract

As complementary metal-oxide-semiconductor (CMOS) transistors approach the nanometer scale, it has become mandatory to incorporate suitable quantum formalism into electron transport simulators. In this work, we present the quantum enhancement of a 2D Multi-Subband Ensemble Monte Carlo (MS-EMC) simulator, which includes a novel module for the direct Source-to-Drain tunneling (S/D tunneling), and its verification in the simulation of Double-Gate Silicon-On-Insulator (DGSOI) transistors and FinFETs. Compared to ballistic Non-Equilibrium Green’s Function (NEGF) simulations, our results show accurate I${}_{D}$ vs. V${}_{GS}$ and subthreshold characteristics for both devices. Besides, we investigate the impact of the effective masses extracted Density Functional Theory (DFT) simulations, showing that they are the key of not only the general thermionic emission behavior of simulated devices, but also the electron probability of experiencing tunneling phenomena.

## 1. Introduction

Conventional and novel transistor architectures have been scaled down in the last decades to achieve better performance and larger integration with both lower power consumption and cost. However, conventional bulk complementary metal-oxide-semiconductor (CMOS) technologies present different problems with scaling, such as short-channel effects, reduction of the mobility, leakage current, degradation of the ON and OFF currents (I${}_{ON}$/I${}_{OFF}$), or variability issues. Novel CMOS transistor architectures, such as Full-Depleted Silicon-On-Insulator (FDSOI) and FinFET, were introduced to mitigate the unwanted effects. Furthermore, in the area of nanotransistor transport simulations, one needs to assess the importance of new phenomena that were not relevant in previous technological nodes [1] in order to explain the electrical behavior of aggressively scaled nanodevices. The simulation of these new phenomena is therefore mandatory to investigate and design the next technology generations and to extend the end of the scaling Roadmap.

At present, different approaches incorporating quantum confinement and tunneling into semi-classical models have become popular due to their modular implementation and reduced computational cost in comparison to purely quantum transport simulation techniques. In particular, the direct Source-to-Drain tunneling (S/D tunneling) starts to play an important role degrading the subthreshold behavior when the channel length is reduced to below 10 nm [2,3], being traditionally considered as a scaling limit in ballistic Non-Equilibrium Green’s Function (NEGF) calculations [4], distorting the MOSFET operation at transistor channel lengths around 3nm [2]. This tunneling mechanism allows electrons to tunnel from the source to the drain through the narrow potential barrier existing between both regions, which is controlled by the gate. As a result, the current is increased, eroding the gate control and the subthreshold slope and increasing the leakage. In the simulation of the direct tunneling phenomena, the employed band structure model must accurately represent the experimental energy gaps and effective masses for the most relevant subbands.

The aim of this work is twofold. First, we will discuss the quantum upgrade of our semi-classical 2D Multi-Subband Ensemble Monte Carlo (MS-EMC) simulation tool [5] through the inclusion of a novel S/D tunneling model. Second, we will perform a comprehensive study of how the effective mass variation in confined channels impacts the transport properties and the S/D tunneling. In particular, we have calibrated our tunneling model against the 2D NEGF solver included in the new simulation environment Nano-Electronic Simulation Software (NESS) [6] bearing in mind that the S/D tunneling is naturally included in the quantum NEGF approach. To understand the impact of electron effective mass variation, the bulk effective masses (m${}_{bulk}$) and calibrated effective masses (m${}_{eff}$) from Density Functional Theory (DFT) are used in the MS-EMC model, which is the preferred technique to calculate the electronic band structure of confined nanostructures.

The paper is organized as follows. Section 2 provides a general overview of the simulation framework, including the outline of the simulated devices (Section 2.1), a brief description of NEGF-NESS (Section 2.2) and MS-EMC tools (Section 2.3). It also reports the S/D tunneling model incorporated in the MS-EMC tool (Section 2.4), together with the effective mass calculation and the corresponding extracted values (Section 2.5). The main findings are presented in Section 3 considering ballistic transport for MS-EMC vs. NEGF comparison (Section 3.1) as well as diffusive simulations for the study of the effective mass variation impact (Section 3.2). Finally, conclusions are drawn in Section 4.

## 2. Simulation Framework and Device Structures

### 2.1. Description of the Simulated Devices

**Figure 1:**
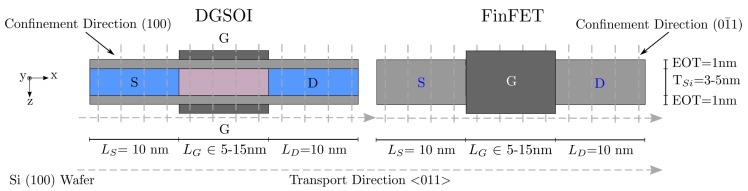
Double-Gate Silicon-On-Insulator (DGSOI) and FinFET structures analyzed in this paper with L${}_{G}$ ranging from 5 to 15 nm and T${}_{Si}$ = 3–5 nm. The 1D Schrödinger equation is solved in the confinement direction for each grid point and the Boltzmann Transport Equation (BTE) is solved by the MC method in the transport plane.

In this work, we have compared two double-gate device architectures, with the main difference related to the confinement direction: a planar Double-Gate Silicon-On-Insulator (DGSOI) transistor and a vertical FinFET. Their description including orientation and design parameters can be found in Figure 1. The corresponding bulk effective masses are summarized in Table 1. The confinement direction for these devices on standard wafers [100] changes from (100) for DGSOI to (0$\bar{1}$1) for FinFET, whereas the transport direction <011> is the same for both. The difference in the confinement direction modifies the electron distribution in the subbands and, consequently, the electrostatic potential profile. In addition, the carrier transport effective mass is also modified [7] as it is shown in Section 2.5.

At this stage, it is important to highlight that, although the FinFET is a 3D structure and our simulation approach is 2D, it has been shown that FinFETs with fin heights much higher than the corresponding thicknesses show similar behavior in all transport regimes when using 2D and 3D simulations [8].

The devices under consideration have been parametrized for gate lengths ranging from 5 to 15 nm. A channel thickness T${}_{Si}$ = 3 nm has been chosen for the MS-EMC vs. NEGF comparison. As for the effective mass variation impact, these devices have been simulated for two different channel thickness T${}_{Si}$ = 5 nm and T${}_{Si}$ = 3 nm in order to capture the effect of the channel thickness reduction. The rest of the technological parameters remains constant: a SiO${}_{2}$ gate oxide with EOT = 1 nm and a metal gate work function of 4.385 eV.

### 2.2. Description of the 2D NEGF Module Inside NESS

The effective-mass real-space Hamiltonian can be expressed as,

(1) $$\begin{array}{cc} E^{\prime }\;\psi \left(x,z,y\right)\; & =\left[-\frac{\hbar^{2}}{2m_{x}}\frac{\partial^{2}}{\partial x^{2}}-\frac{\hbar^{2}}{2m_{z}}\frac{\partial^{2}}{\partial z^{2}}+\frac{\hbar^{2}k_{y}^{2}}{2m_{y}}+V\left(x,y,z\right)\right]\psi \left(x,z,y\right), \end{array}$$

assuming *y*-direction as the periodic transverse direction. The total energy $E^{\prime }$ can also be written as $E^{\prime }=E_{x}+\frac{\hbar^{2}k_{y}^{2}}{2m_{y}}$, where $E_{x}$ is the electron energy in the transport direction. The Hamiltonian in Equation (1) is then transformed to the mode-space representation in order to reduce the computational cost of quantum transport simulations [9]. This was carried out by means of a recursive NEGF approach [10] as implemented in NESS [6] to extract the most relevant physical quantities such as the carrier charge and current. Further, we briefly summarize the main expressions of the NEGF formalism.

For 2D devices and by exploiting the effective-mass approximation, all the transverse modes $k_{y}$ can be treated as independent devices in parallel. Then, within the ballistic regime and under steady-state conditions, the retarded and lesser Green’s function for the active device region are written, in matrix notation, as:

(2) $$\begin{array}{cc} G^{R}\left(E_{x}\right) & ={\left[E_{x}I-{\tilde{H}}_{M}-\Sigma_{S}^{R}\left(E_{x}\right)-\Sigma_{D}^{R}\left(E_{x}\right)\right]}^{-1}, \end{array}$$

(3) $$\begin{array}{cc} G^{<}\left(E_{x}\right) & =G^{R}\left(E_{x}\right)\left[\Sigma_{S}^{<}\left(E_{x}\right)+\Sigma_{D}^{<}\left(E_{x}\right)\right]G^{R†}\left(E_{x}\right), \end{array}$$

where $H_{M}$ and $\Sigma_{C}^{R/<}$ are the Hamiltonian and the retarded/lesser contact self-energies (C=S/D) in the mode-space representation, respectively. The retarded Green’s function at the contacts $G^{R}=g_{C}^{R}$ is calculated by means of the Sancho-Lopez-Rubio recursive method [11], allowing straightforwardly the evaluation of $\Sigma_{C}^{R}$ as

(4) $$\Sigma_{C}^{R}\left(E_{x}\right)=t_{M}\cdot g_{C}^{R}\left(E_{x}\right)\cdot t_{M}^{†}$$

where the mode-space hopping parameters $t_{M}$ are computed as in Ref. [9]. The lesser contact self-energy $\Sigma_{C}^{<}$ can be then computed from:

(5) $$\begin{array}{c} \Sigma_{C}^{<}\left(E_{x}\right)=-F_{S/D}\left(E_{x}\right)\left(g_{C}^{R}\left(E_{x}\right)-g_{C}^{R†}\left(E_{x}\right)\right). \end{array}$$

with

(6) $$\begin{array}{c} F_{S/D}\left(E_{x}\right)=\frac{L_{y}}{2\pi }\int dk_{y}f_{S/D}\left(E_{x}+\frac{\hbar^{2}k_{y}^{2}}{2m_{y}}\right), \end{array}$$

where $f_{S/D}$ is the Fermi-Dirac distribution and $L_{y}$ is the periodic length in *y*-direction. Finally, the 3D carrier concentration and current are calculated in the mode-space representation as follows:

(7) $$\begin{array}{cc} n(x_{i},y,z_{j}) & =-\frac{i}{\Delta x_{i}\Delta z_{j}L_{y}}\sum_{nm}\int \frac{dE_{x}}{2\pi }\phi_{n}\left(z_{j}\right)G_{nm}^{<}\left(x_{i},x_{i};E_{x}\right)\phi_{m}^{*}\left(z_{j}\right), \end{array}$$

(8) $$\begin{array}{cc} I(x_{i}) & =-\frac{2e}{\hbar L_{y}}\int \frac{dE_{x}}{2\pi }\mathrm{Tr}\left[t_{M}\left(i\right)G^{<}\left(x_{i+1},x_{i};E_{x}\right)-G^{<}\left(x_{i},x_{i+1};E_{x}\right)t_{M}^{†}\left(i\right)\right]. \end{array}$$

where $\phi_{n}\left(z\right)$ is the confinement wave-function for the subband *n*, whereas, matrices $t_{M}\left(i\right)$ couple two successive layers. Finally, Equations (1) to (3), (5) and (7) are solved self-consistently with Poisson’s equation.

### 2.3. General Overview of the 2D MS-EMC Tool

The 2D MS-EMC simulation framework [5] used in this work is based on a decoupled mode-space quantum transport [12] and a semi-classical approach. The simulator solves the Schrödinger equation in the discretization slices along the confinement direction and the Boltzmann Transport Equation (BTE) in the transport plane (Figure 1). Both equations are coupled through the 2D Poisson equation in the whole 2D simulation domain to keep the self-consistency of the solution. This tool has been widely used in different scenarios including the study of different tunneling mechanisms in similar devices [13]. Due to the modular design of our MS-EMC tool, the inclusion of these tunneling phenomena can be successfully included via additional modules that treat them as separate transport mechanisms without increasing the computational time in comparison to purely quantum simulators. These modules can be switched on or off depending on the simulation scenario, offering the possibility of studying each tunneling mechanism independently.

### 2.4. Description of the S/D Tunneling Model Inside the 2D MC-EMC Tool

S/D tunneling has been included as a separated transport mechanism in the 2D MS-EMC tool described in Section 2.3. It has been implemented as a stochastic mechanism evaluated for each superparticle at the end of the Monte Carlo cycle [14]. When this tunneling mechanism is considered, an electron near the S/D potential barrier will be either reflected or transmitted through it.

The first step is to calculate the tunneling probability by the Wentzel-Kramers-Brillouin (WKB) approximation [15]. It mainly depends on the energy and position of the carrier in the device; the transport effective mass (namely $m_{x}$ in Table 1 and Table 2); and the energy profile of the *i*-th subband determining the shape of the tunneling barrier ($E_{i}\left(x\right)$), which is calculated as a solution of the 1D Schrödinger equation. The probability of tunneling through the barrier is equivalent to the transmission coefficient, and determines the fraction of electrons experiencing S/D tunneling at a given energy below the potential barrier. The tunneling probability of the electron for a given energy (T${}_{WKB}$(E${}_{x}$)) is:

(9) $$T_{WKB}\left(E_{x}\right)=\exp\left\{-\frac{2}{\hbar }\int_{a}^{b}\;\sqrt{2m_{x}\left(E_{i}\left(x\right)-E_{x}\right)}\;dx\right\},$$

where *a* and *b* are the limits of the tunneling path, and $E_{x}$ is the total energy in the transport plane considering only the projection of the kinetic energy in the direction that faces the potential barrier.

It has been reported for the short-gate length devices that this model overesitimated the number of superparticles experiencing S/D tunneling compared to NEGF approach [16]. This model was compared to NEGF simulations showing an overestimation of the number of superparticles experiencing S/D tunneling, especially for short-gate length devices. In order to reduce this discrepancy, the tunneling model in Equation (9) has been reformulated following a non-local WKB probability approach as stated in Appendix B of Ref. [17]. In the context of a 2D simulation domain, the new S/D tunneling probability for a given energy (T${}_{DT}$(E${}_{x}$)) is now defined as:

(10) $$T_{DT}\left(E_{x}\right)=\frac{\Delta_{y}}{2\sqrt{\pi }}{\left[\hbar \int_{a}^{b}\frac{dx}{\sqrt{2m_{x}\left(E_{i}\left(x\right)-E_{x}\right)}}\right]}^{-1/2}\cdot T_{WKB}\left(E_{x}\right),$$

where $\Delta_{y}$ is the mesh spacing in the direction normal to transport. As this direction is not taken into account in our 2D MS-EMC tool, the value of $\Delta_{y}$ has been calibrated to fulfill the following conditions: *(i)* the force T${}_{DT}$(E${}_{x}$) to be in the range [0–1]; *(ii)* to be small enough to be consistent with the periodic boundary condition in the y direction; and *(iii)* to have similar degradation in the subthreshold region compared to NEGF calculations for the device with L${}_{G}$ = 7.5 nm (shown in Section 3.1). In order to assess the S/D tunneling impact as a function of the gate length, $\Delta_{y}$ has been calculated according to the mesh spacing in the transport direction ($\Delta_{x}$). A fixed number of mesh points is taken in our calculation in the transport direction regardless of the gate length of the considered device, so that $\Delta_{x}$ varies as L${}_{G}$ does so. In this particular study, we have chosen $\Delta_{y}$ = 0.05$\Delta_{x}$, which corresponds to $\Delta_{y}$ = 0.01 nm for the device with L${}_{G}$ = 7.5 nm.

The second step is to determine whether the particle tunnels or not by using a rejection criterion. To do so, a uniformly distributed random number r${}_{DT}$ is generated between 0 and 1 and compared to T${}_{DT}$(E${}_{x}$). If r${}_{DT}$ ≤ T${}_{DT}$(E${}_{x}$), the superparticle will cross the barrier; otherwise, it will turn back suffering a back-scattering. Finally, if the superparticle undergoes S/D tunneling, its motion inside the potential barrier is described using Newton’s mechanics considering an inverted potential profile and ballistic transport [18].

### 2.5. Description of the Effective Mass Calculation

To adopt more reasonable conduction band structures in nanoscaled structures, we accurately calculate m${}_{eff}$ by using DFT implemented in QuantumATK tool of Synopsys [19]. Table 2 summarizes the values of the masses for both devices (DGSOI and FinFET) studied here. It is also important to highlight at this point that the lowest energy subband changes from $\Delta_{2}$ in the planar transistor to $\Delta_{4}$ in the vertical one.

**Figure 2:**
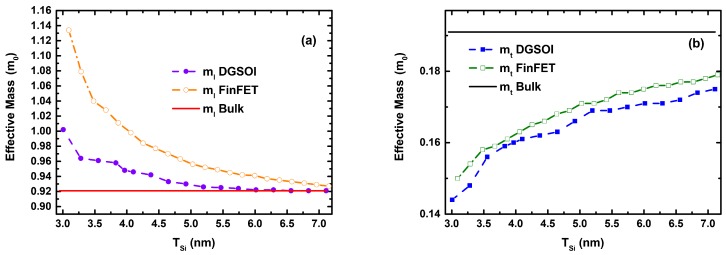
(**a**) Longitudinal ($m_{l}$) and (**b**) transverse ($m_{t}$) effective masses calculated using Density Functional Theory (DFT) as well as the bulk effective masses as a function of the silicon thickness (T${}_{Si}$) for DGSOI ((100) Confinement Orientation) and FinFET ((0$\bar{1}$1) Confinement Orientation) devices.

**Figure 3:**
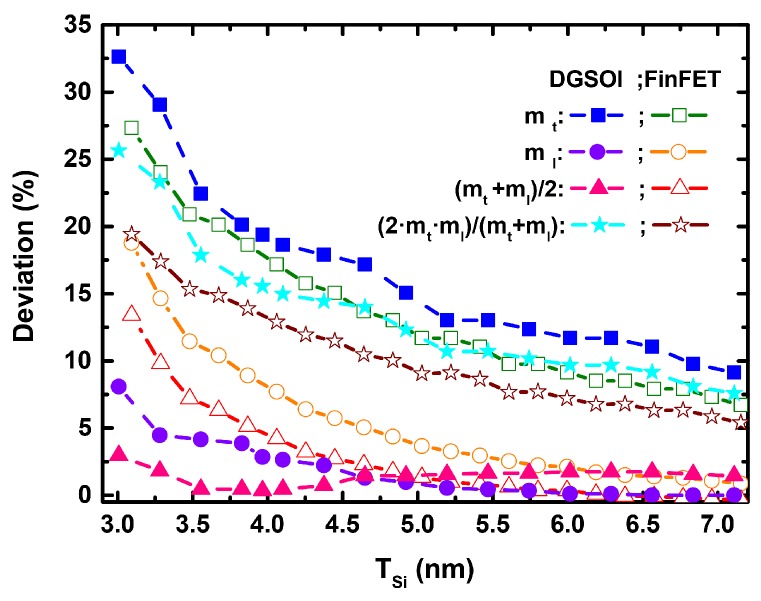
Deviation (%) of the longitudinal ($m_{l}$) and transverse ($m_{t}$) effective masses and their combinations needed in Table 2 as a function of the silicon thickness (T${}_{Si}$) for DGSOI ((100) confinement orientation) as well as FinFET ((0$\bar{1}$1) confinement orientation) devices.

Figure 2 shows the difference of the longitudinal ($m_{l}$) and transverse ($m_{t}$) effective masses calculated as a function of the silicon thickness (T${}_{Si}$) for the two different confinement orientations. It is clearly shown that the effective masses tend to m${}_{bulk}$ for larger T${}_{Si}$. Although these masses ($m_{l}$ and $m_{t}$) are included in the 2D MS-EMC tool as input parameters, it is important to analyze the modification of $m_{x}$ (transport mass), $m_{z}$ (confinement mass) and $m_{y}$ (mass in the direction normal to transport). Their expressions are shown in Table 1, and their particular values are grouped in Table 2 for the two T${}_{Si}$ values herein considered. In order to study the impact of T${}_{Si}$ reduction, the deviations (in %) of $m_{l}$, $m_{t}$ and their combinations included in Table 1 have been calculated (Figure 3) as $100\cdot \left|m_{bulk}-m_{eff}\right|/m_{eff}$. It is interesting to mention that the deviation in m${}_{t}$ is more noticeable than that of (m${}_{t}$ + m${}_{l}$)/2, which corresponds to $m_{x}$ in the S/D tunneling model for the fundamental subband of the planar and vertical devices, respectively. In particular, they drop from ∼35% (DGSOI devices) to ∼15% (FinFETs) for T${}_{Si}$ = 3 nm and from ∼17.5% (DGSOI devices) to ∼2.5% (FinFETs) for T${}_{Si}$ = 5 nm.

## 3. Simulation Results and Discussions

### 3.1. Comparison of MS-EMC with S/D Tunneling Models vs. NEGF

**Figure 4:**
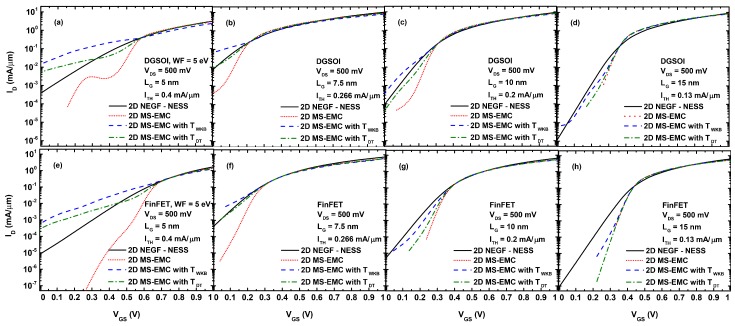
I${}_{D}$ vs. V${}_{GS}$ in the DGSOI and FinFET devices at V${}_{DS}$ = 500 mV with L${}_{G}$ 5 nm (**a**,**e**), 7.5 nm (**b**,**f**), 10 nm (**c**,**g**), and 15 nm (**d**,**h**), considering the four types of simulations are: *(1)* Non-Equilibrium Green’s Function (NEGF) approach in the Nano-Electronic Simulation Software (NESS) tool, *(2)* Multi-Subband Ensemble Monte Carlo (MS-EMC) tool without any type of tunneling, and MS-EMC tool with the Source-to-Drain tunneling (S/D tunneling) module using *(3)* T${}_{WKB}$(E${}_{x}$) and *(4)* T${}_{DT}$(E${}_{x}$).

The I${}_{D}$ vs. V${}_{GS}$ characteristics obtained from ballistic simulations of the DGSOI and FinFET devices at V${}_{DS}$ = 500 mV with gate length ranging from 5 nm to 15 nm are shown in Figure 4. Four types of simulations are displayed: *(1)* the NEGF approach in the NESS tool, *(2)* the MS-EMC tool without any type of tunneling, and the MS-EMC tool with the S/D tunneling module using *(3)* T${}_{WKB}$(E${}_{x}$) and *(4)* T${}_{DT}$(E${}_{x}$). In order to attain a good I${}_{ON}$/I${}_{OFF}$ behavior, the work function for the devices with Lg = 5 nm was chosen to be 5 eV rather than 4.385 eV, as for the rest of cases. In general, the I${}_{D}$ vs. V${}_{GS}$ characteristics were shifted to have the same threshold current (I${}_{TH}$), showing similar I${}_{ON}$ in all the cases. The particular values of I${}_{TH}$ as a function of the gate length are included in Figure 4 too.

Regarding the OFF region, where S/D tunneling was more noticeable, we can reach the following conclusions when the MS-EMC results are compared against NEGF. First, the simulation without any tunneling reduced I${}_{OFF}$ substantially due to the absence of particles below the barrier. Second, there was an overestimation of I${}_{OFF}$ when the tunneling probability was calculated by T${}_{WKB}$(E${}_{x}$), specially for L${}_{G}\leq$ 10 nm. Third, when the tunneling probability was chosen as T${}_{DT}$(E${}_{x}$), the current was comparable to NEGF, showing a reduction of I${}_{OFF}$. In particular for L${}_{G}$ = 7.5 nm, it was really similar to NEGF because, as anticipated earlier, the parameter $\Delta_{y}$ included in Equation (10) was calibrated against the NEGF results. Fourth, the S/D tunneling was important in ultra-scaled devices with L${}_{G}\leq$ 10 nm due to the dimensions of the potential barrier. Consequently, for L${}_{G}$ = 15 nm, there was almost no difference in the OFF current among the different MS-EMC cases. Moreover, the inherent statistical nature of the MC method also manifested in Figure 4a by the fluctuations in the subthreshold regime for the simulation without any tunneling module.

**Figure 5:**
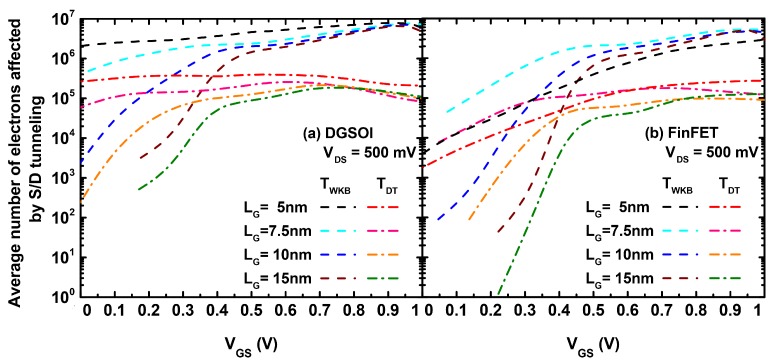
Average number of electrons (in arbitrary units) affected by S/D tunneling as a function of the V${}_{GS}$ in the (**a**) DGSOI and (**b**) FinFET devices at V${}_{DS}$ = 500 mV with L${}_{G}$ = 5, 7.5, 10, and 15 nm, for the MS-EMC tool with the S/D tunneling module using T${}_{WKB}$(E${}_{x}$) and T${}_{DT}$(E${}_{x}$).

Figure 5 shows the average number of electrons affected by S/D tunneling for the simulations considered in Figure 4. In general, the drain current in Monte Carlo simulators was calculated by the spatial average of the electron current along the channel. Therefore, the number of electrons located inside the potential barrier due to the S/D tunneling model contributed to the increase of the total current. On the other hand, as depicted in Figure 5, the T${}_{DT}$(E${}_{x}$) probability reduced the number of electrons crossing the potential barrier compared to the T${}_{WKB}$(E${}_{x}$) case and thus there was a reduction of I${}_{OFF}$. It is also worth to mention that the number of electrons affected by S/D tunneling approached the same value at high V${}_{GS}$ regime for both approaches (T${}_{WKB}$(E${}_{x}$) and T${}_{DT}$(E${}_{x}$)) and for both devices regardless of the gate length.

The subthreshold swing (SS) is one of the main parameters used to determine the behavior of electronic devices in the OFF region. In practice, the best MOSFET implementations cannot reduce SS < 60 mV/dec. For the SS calculation, we have considered the I${}_{D}$ decade between 10${}^{-3}$ mA/μm and 10${}^{-2}$ mA/μm (or between 10${}^{-2}$ mA/μm and 10${}^{-1}$ mA/μm) in order to avoid the stochastic noise of the Monte Carlo simulations at very low V${}_{GS}$.

**Figure 6:**
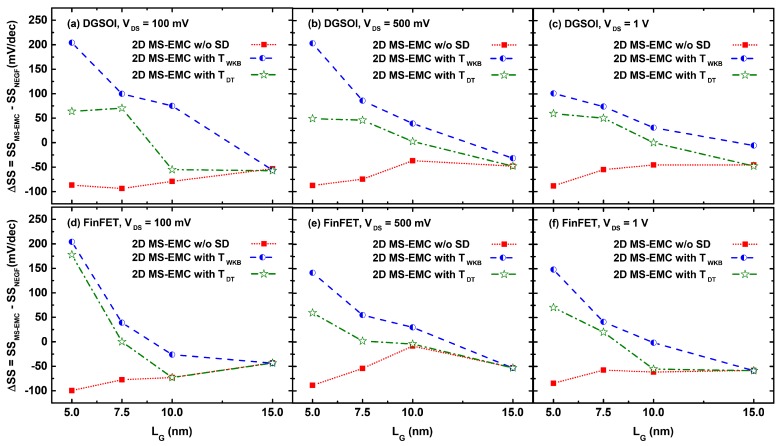
ΔSS as a function of the gate length in the DGSOI and FinFET devices at V${}_{DS}$ = 100 mV (**a**,**d**), V${}_{DS}$ = 500 mV (**b**,**e**), and V${}_{DS}$ = 1 V (**c**,**f**), calculated as the difference between the 2D NEGF-NESS and the 2D MS-EMC tools considering the three combinations: a simulation without any tunneling module and both S/D tunneling modules with T${}_{WKB}$(E${}_{x}$) and T${}_{DT}$(E${}_{x}$) probabilities.

Figure 6 shows the SS difference (ΔSS) between MS-EMC and NEGF simulations. In general, and for all values of V${}_{DS}$, we have reached the following three conclusions. First, ΔSS was negative for the MC simulation without any tunneling due to its lower I${}_{OFF}$ compared to NEGF case. Second, ΔSS tended to zero for the FinFET device with L${}_{G}\geq$ 7.5 nm, showing the excellent agreement between both approaches for that device. Third, for L${}_{G}$ = 15 nm, ΔSS was again negative due to the lower I${}_{OFF}$ of the different MS-EMC simulations.

### 3.2. Impact of the Effective Mass Choice

**Figure 7:**
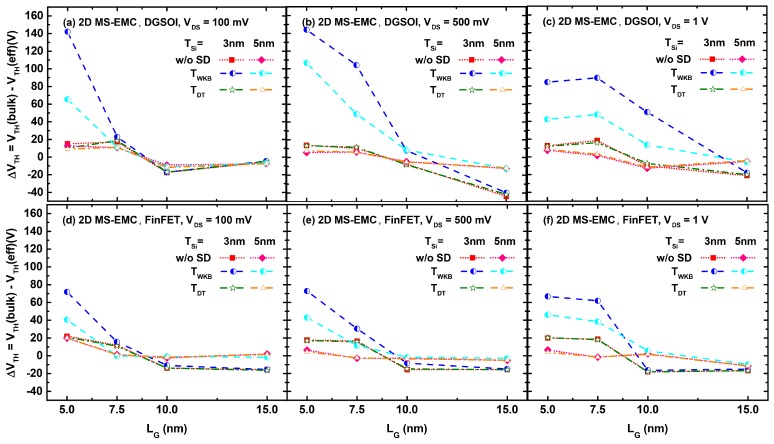
ΔV${}_{TH}$ as a function of the gate length in the DGSOI and FinFET devices with silicon thickness T${}_{Si}$ = 3–5 nm at V${}_{DS}$ = 100 mV (**a**,**d**), V${}_{DS}$ = 500 mV (**b**,**e**), and V${}_{DS}$ = 1 V (**c**,**f**), considering the three 2D MS-EMC combinations: without any tunneling module and with both S/D tunneling modules using T${}_{WKB}$(E${}_{x}$) and T${}_{DT}$(E${}_{x}$) probabilities.

In general, the utilization of m${}_{eff}$ instead of m${}_{bulk}$ results in a shift of the I${}_{D}$ vs. V${}_{GS}$ characteristics ([20]). Accordingly, we have focused on the study of this impact on the threshold voltage shift (ΔV${}_{TH}$) calculated as the difference of V${}_{TH}$ using m${}_{eff}$ and m${}_{bulk}$ (Figure 7). $V_{TH}$ has been calculated in this work using the constant drain current method [21]. In this section, non-equilibrium simulations (including acoustic phonon, optical, phonon, surface roughness, and Coulomb scattering mechanisms) have been considered using the 2D MS-EMC tool with the three possible combinations: *(1)* without any tunneling module, *(2)* with S/D tunneling using T${}_{WKB}$(E${}_{x}$), and *(3)* with S/D tunneling using T${}_{DT}$(E${}_{x}$).

Four observations can be made based on the ΔV${}_{TH}$ curves displayed in Figure 7. First, ΔV${}_{TH}$ was positive for the ultra-scaled devices (L${}_{G}$ = 5 and 7.5 nm) because the use of m${}_{eff}$ increases the current, whereas the opposite trend (ΔV${}_{TH}$ < 0) was observed for devices with L${}_{G}\geq$ 10 nm. Second, ΔV${}_{TH}$ was reduced for thicker devices (T${}_{Si}$ = 5 nm) because m${}_{eff}$ tends to m${}_{bulk}$ when T${}_{Si}$ increases. Third, a similar behavior is shown in Figure 7 for the simulations without any tunneling and with T${}_{DT}$(E${}_{x}$). However, when the tunneling probability was calculated using T${}_{WKB}$(E${}_{x}$) the difference between using m${}_{eff}$ instead of m${}_{bulk}$ is greater due to the overestimation of the superparticles experiencing S/D tunneling. This effect became more relevant when the device size is reduced. In fact, this influence was significant for L${}_{G}$ = 5 nm at all V${}_{DS}$ and it was extended to longer devices as V${}_{DS}$ increased. Fourth, the impact of the effective mass choice was smaller in the FinFET compared to the DGSOI device due to the lower deviation of its effective transport mass (m${}_{x}$) shown in Figure 3.

## 4. Conclusions

This work presents the quantum enhancement of a semi-classical 2D MS-EMC simulator and its application to DGSOI transistors and FinFETs. It has been demonstrated as a useful tool for the optimization of devices targeting sub-10 nm nodes thanks to its higher computational efficiency. Two different approaches to consider S/D tunneling in MC are described in this work and their results with FinFET and DGSOI are compared to those from NEGF formalism. One of these models needs to be calibrated against quantum transport simulations. Results obtained from the MS-EMC code show an excellent agreement with the NEGF simulations in the subthreshold region. The impact of realistic effective masses, calculated from first principles, on electron transport has also been studied by means of MS-EMC simulations. Our findings suggest that effective masses variation alters in a significant way the tunneling probability in the subthreshold regime, in agreement with reported results in the literature. 

## Figures and Tables

**Table 1:** Silicon bulk effective masses (m${}_{bulk}$) for the different crystallographic directions considered in the DGSOI and FinFET devices. Herein, $m_{x}$ and $m_{z}$ are the transport and confinement masses, respectively; $m_{y}$ is the effective mass in the periodic transverse direction; m${}_{0}$ is the free electron mass; and the subindex of Δ represents the degeneracy factor associated with the conduction band valley.

Device	Valley	m${}_{\mathrm{bulk}}$		
		$m_{x}$	$m_{y}$	$m_{z}$
DGSOI	$\Delta_{2}$	$m_{t}$ = 0.193 m${}_{0}$	$m_{t}$ = 0.193 m${}_{0}$	$m_{l}$ = 0.912 m${}_{0}$
(100)<011>	$\Delta_{4}$	$\frac{2m_{l}m_{t}}{m_{l}+m_{t}}$ = 0.319 m${}_{0}$	$\frac{m_{l}+m_{t}}{2}$ = 0.553 m${}_{0}$	$m_{t}$ = 0.193 m${}_{0}$
FinFET	$\Delta_{2}$	$m_{t}$ = 0.193 m${}_{0}$	$m_{l}$ = 0.912 m${}_{0}$	$m_{t}$ = 0.193 m${}_{0}$
(0$\bar{1}$1)<011>	$\Delta_{4}$	$\frac{m_{l}+m_{t}}{2}$ = 0.553 m${}_{0}$	$m_{t}$ = 0.193 m${}_{0}$	$\frac{2m_{l}m_{t}}{m_{l}+m_{t}}$ = 0.319 m${}_{0}$

**Table 2:** Effective masses (m${}_{eff}$) considering the DGSOI and FinFET devices herein studied with silicon thickness T${}_{Si}$ = 3–5 nm using DFT simulations included in QuantumATK of Synopsys [19]. Notice that $m_{x}$ and $m_{z}$ are the transport and confinement masses, respectively, $m_{y}$ is the mass in the direction normal to transport, m${}_{0}$ is the free electron mass, and the subindex of Δ represents the degeneracy factor associated with the conduction band valley.

Device	Valley	T${}_{\mathrm{Si}}$ = 3 nm			T${}_{\mathrm{Si}}$ = 5 nm		
		$m_{x}$	$m_{y}$	$m_{z}$	$m_{x}$	$m_{y}$	$m_{z}$
DGSOI	$\Delta_{2}$	0.144 m${}_{0}$	0.144 m${}_{0}$	1.002 m${}_{0}$	0.166 m${}_{0}$	0.166 m${}_{0}$	0.93 m${}_{0}$
(100)<011>	$\Delta_{4}$	0.252 m${}_{0}$	0.573 m${}_{0}$	0.144 m${}_{0}$	0.282 m${}_{0}$	0.548 m${}_{0}$	0.166 m${}_{0}$
FinFET	$\Delta_{2}$	0.15 m${}_{0}$	1.134 m${}_{0}$	0.15 m${}_{0}$	0.171 m${}_{0}$	0.956 m${}_{0}$	0.171 m${}_{0}$
(0$\bar{1}$1)<011>	$\Delta_{4}$	0.642 m${}_{0}$	0.15 m${}_{0}$	0.265 m${}_{0}$	0.563 m${}_{0}$	0.171 m${}_{0}$	0.29 m${}_{0}$
